# A biomechanical analysis of ischiofemoral impingement in a cadaver model

**DOI:** 10.1093/jhps/hnaa052

**Published:** 2020-12-02

**Authors:** Yoichi Murata, Naomasa Fukase, Hajime Utsunomiya, Alex W Brady, Samuel I Rosenberg, Patrick Quinn, Soshi Uchida, Hal David Martin, Marc J Philippon

**Affiliations:** 1 The Steadman Philippon Research Institute and the Steadman Clinic, Vail, CO, USA; 2 Department of Orthopaedic Surgery and Sports Medicine, Wakamatsu Hospital of University of Occupational and Environmental Health, Kitakyushu, Japan; 3 Baylor Scott & White Hip Preservation Center at Baylor University Medical Center

## Abstract

Ischiofemoral impingement (IFI) occurs due to the diminishing of space between the ischium and lesser trochanter. During a robotic hip study, one hip presented with indications of IFI, an opportunity to explore the pathophysiology and treatment strategies for this unusual condition. This specimen underwent kinematic tests in two states: (i) native lesser trochanter and (ii) resected lesser trochanter. The ‘Resected lesser trochanter’ state was found to increase the hip range of motion and decrease femoral head translation by eliminating contact between the femur and pelvis. These results suggest that lesser trochanteric resection would provide physical benefit for IFI patients.

## CASE REPORT

Ischiofemoral impingement (IFI) was visually observed during adduction, extension and external rotation testing, and the specimen was used for evaluating IFI biomechanics. One cadaveric hip joint from a 54-year-old female with a body mass index (BMI) of 28.9 kg/m^2^ and no previous history of hip injury or surgical procedure was studied. The purpose of this study was to compare the biomechanics before and after lesser trochanteric resection. All soft tissue superficial to the hip capsule was removed. The setup and coordinate frames of the pelvis and femoral head were stated in previous literature.^1^ Using a three-dimensional coordinate measuring machine (Romer Absolute Arm, Hexagon Metrology, Surrey, Great Britain), the relevant bony anatomy of the specimen was digitized to construct a joint coordinate system. The neutral position of the hip was obtained prior to mounting, according to the International Society of Biomechanics standard.^2^ Next, the femur and pelvis were individually potted in cylindrical molds of polymethyl methacrylate. The joint was rigidly fixed to a six-degrees-of-freedom robotic system (Kuka KR-60, Augsberg, Germany) by securing the pelvis to the robot end effector and the femur to a rigid pedestal. The pedestal was equipped with a six-axis universal force/torque sensor (ATI). The software simVITRO was utilized to control the robot and prescribe the desired forces and motions to the joint. To obtain the resected lesser trochanter state, osteotomy through the lesser trochanter was performed using a sagittal saw. The resected lesser trochanter size was 24.7 mm × 14.9 mm × 8.6 mm.

**Figure 1.**
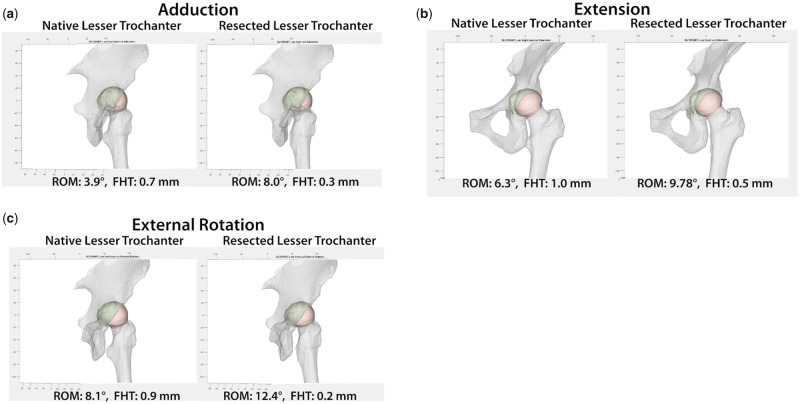
A comparison of ROM and FHT between “Native Lesser Trochanter” and “Resected Lesser Trochanter” in (**a**) Adduction, (**b**) Extension, and (**c**) External rotation. ROM: range of motion; FHT: femoral head translation.

The specimen underwent three kinematic range of motion (ROM) tests: (i) Adduction, (ii) Extension and (iii) External Rotation. All tests were performed in two sequential states: (i) Native lesser trochanter and (ii) Resected lesser trochanter. Forces were minimized in the anterior and lateral directions while a 60 N superior compressive force was applied to seat the joint. A 5 Nm torque was applied to the tested rotational axis while two other rotational axes were maintained at 0 degrees in position control. For all tests, ROM (degrees) and femoral head translation (FHT) (millimeters) were recorded. When compared to the ‘Native lesser trochanter’ state, increases in ROM were observed in the ‘Resected lesser trochanter’ state. Compared to the ‘Native lesser trochanter’ state, decreases in FHT were observed in the ‘Resected lesser trochanter’ state (Fig. 1).

## DISCUSSION

This is the first biomechanical report indicating that the lesser trochanter directly causes IFI, decreases ROM and increases FHT. Gollwitzer *et al*. discovered correlations between specific IFI symptoms, magnetic resonance (MR) imaging, surgical treatment and non-surgical care.^3^ Several reports have shown that lesser trochanteric resection can lead to favorable clinical outcomes.^4^^,^^5^ This study supports the previous articles; lesser trochanteric osteotomy is an effective way to increase ROM while decreasing FHT. As this study only presents a single case of IFI, further exploration and understanding of hip biomechanics in IFI patients is essential for the development of adequate treatment. In conclusion, lesser trochanteric resection could provide effective results for IFI patients.

## Ethical approval

Institutional review board approval was not required as a result of the fact that de-identified human cadaveric specimens are exempt from review consideration at our institution.
